# An exploratory study of community factors relevant for participatory malaria control on Rusinga Island, western Kenya

**DOI:** 10.1186/1475-2875-6-48

**Published:** 2007-04-24

**Authors:** Pamela Opiyo, W Richard Mukabana, Ibrahim Kiche, Evan Mathenge, Gerry F Killeen, Ulrike Fillinger

**Affiliations:** 1 SNV Netherlands Development Organisation, P.O Box 410576, Kasama, Zambia; 2 School of Biological Sciences, University of Nairobi, P.O. Box 30197-00100 GPO, Nairobi, Kenya; 3 Christian Children's Fund (CCF), Nairobi, Kenya; 4 Ifakara Health Research and Development Centre, Public Health Entomology Unit, PO Box 78373, Dar es Salaam, Tanzania; 5 Swiss Tropical Institute, Department of Public Health and Epidemiology, Socinstrasse 57, Basel, CH-4002, Switzerland; 6 Durham University, School of Biological and Biomedical Sciences, South Road, Durham DH1 3LE, UK

## Abstract

**Background:**

Capacity strengthening of rural communities, and the various actors that support them, is needed to enable them to lead their own malaria control programmes. Here the existing capacity of a rural community in western Kenya was evaluated in preparation for a larger intervention.

**Methods:**

Focus group discussions and semi-structured individual interviews were carried out in 1,451 households to determine (1) demographics of respondent and household; (2) socio-economic status of the household; (3) knowledge and beliefs about malaria (symptoms, prevention methods, mosquito life cycle); (4) typical practices used for malaria prevention; (5) the treatment-seeking behaviour and household expenditure for malaria treatment; and (6) the willingness to prepare and implement community-based vector control.

**Results:**

Malaria was considered a major threat to life but relevant knowledge was a chimera of scientific knowledge and traditional beliefs, which combined with socio-economic circumstances, leads to ineffective malaria prevention. The actual malaria prevention behaviour practiced by community members differed significantly from methods known to the respondents. Beside bednet use, the major interventions implemented were bush clearing and various hygienic measures, even though these are ineffective for malaria prevention. Encouragingly, most respondents believed malaria could be controlled and were willing to contribute to a community-based malaria control program but felt they needed outside assistance.

**Conclusion:**

Culturally sensitive but evidence-based education interventions, utilizing participatory tools, are urgently required which consider traditional beliefs and enable understanding of causal connections between mosquito ecology, parasite transmission and the diagnosis, treatment and prevention of disease. Community-based organizations and schools need to be equipped with knowledge through partnerships with national and international research and tertiary education institutions so that evidence-based research can be applied at the grassroots level.

## Background

Malaria imposes a huge burden upon the health and economic development of tropical nations [1-3] and has been identified as a major obstacle towards achieving several of the health-related Millennium Development Goals [3,4]. The disease causes widespread premature death and suffering, imposes financial hardship on poor households, retards economic growth and undermines living standards. The vast majority of the world's malaria burden rests in sub-Saharan Africa (SSA) [5] where it is directly responsible for one in five childhood deaths and indirectly contributes to a sizeable proportion of childhood morbidity and mortality resulting from additional illnesses such as respiratory infections, diarrhoeal diseases, iron-deficiency anaemia and malnutrition [4]. An estimated 8.2 million cases of malaria are reported in Kenya every year, out of a total population of 30 million. In Kenya alone, malaria kills an average of 72 children under five years of age each day [6]. The economic burden of malaria for households can be extremely high. Treatment costs for small-scale farmers in rural Kenya have been estimated to be as high as 7% of the monthly household expenditure [6], not considering any costs for prevention measures.

Malaria risk and disease burden is inequitably distributed, not only at global and regional levels but also at household level because poor housing, lack of education and access to healthcare services create a vicious cycle of enhanced vulnerability to malaria due to increased exposure, high household medical costs, reduced ability to pay for treatment, and so on [6,7]. Decisions for prevention or treatment are made depending on economic ability of the household, perceived susceptibility and assessment of consequences. Furthermore, malaria transmission is often facilitated because environmental degradation, poor drainage and clearing of vegetation readily promote the proliferation of mosquito species such as *Anopheles gambiae *which propagates itself in sunlit, transient water bodies, notably artificial habitats associated with human activities [8-12]. Malaria, poverty and environmental change are inextricably linked and remain closely associated across most of Africa [13].

Rural areas have always been a major challenge for disease control worldwide, but the involvement and active participation of communities has been identified as a key factor for success in these environments [14-18]. Malaria remains robustly endemic in most rural communities of SSA so a central aim of the Roll Back Malaria Partnership (RBMP) is to strengthen the local capacities of communities to identify malaria as one of their main health problems and then take the lead in developing and implementing solutions to these problems in partnership with different actors such as non-governmental organizations providing organizational support and research institutions acting as technical consultants [19-24]. In the past, malaria was predominantly viewed only as a biomedical problem, however, successful disease control at the community level needs to take the human behaviour, socio-cultural and economic context into account in order to successfully impact the disease through active participation and changing of risk behaviours [22,25]. These factors, together with the experienced obstacles of earlier vertical, top-down malaria eradication programs, have contributed to the current emphasis on community-based strategies [22,26].

Although, considerable difficulties have been reported in conducting community-based disease control [27-30] there is a large evidence base where such horizontal approaches have been successful because of a true partnership between the community and programme staff. Key elements of these programmes are the generation of a feeling of empowerment, local ownership and responsibility [19,31] and the application of action-oriented and participatory approaches [23,32]. Extended project periods beyond the usual research cycles of three to five years are also necessary to establish [23] and evaluate community-based interventions since the modification of attitude and behaviour may take years to accomplish [28]. Successful examples of programmes with community participation include the control of dengue [28,31], dracunculiasis [33,34], onchocerciasis [35] and urinary schistosomiasis [36,37]. In malaria control, specifically in Africa, few of the projects have been truly 'bottom up' community initiated like the Saradidi Rural Health Development Programme, Kenya [38], but the term is widely used to refer to community co-operation or acceptance of schemes introduced through health education from outside and reflecting national priorities and targets [23,39]. The vast majority of projects with community involvement target the improvement of treatment-seeking behaviour, access to prompt diagnosis and treatment through training of community health workers and shop keepers [26,27,40-46] and the distribution and coverage with insecticide-treated nets (ITNs) [43,47]. Relatively few projects show community-led vector control that goes beyond personal protection measures e.g. environmental modifications and larviciding [48-52].

The study presented here was implemented on Rusinga Island in Lake Victoria, Suba District, western Kenya as part of the Rusinga Malaria Project (RMP) of the Rusinga Island Child & Family Programme (RICFP), a local community based organization (CBO) affiliated to the international non-governmental (NGO) organization Christian Children's Funds – Kenya (CCF-K). Here community members organized in the RICFP identified malaria as a major threat in their daily life and felt the need to take action to reduce the malaria burden on the island. As a consequence, community members and staff of CCF-K approached locally-based researchers for assistance in their fight against malaria on Rusinga, acknowledging that the CBO's (and NGO's) knowledge on how to tackle the problem was insufficient [20].

The authors propose that a community-implemented malaria control programme can only be successful and, even more importantly, sustainable if the community considers malaria to be one of their major problems and have the knowledge and skills to participate in its prevention and programme evaluation. The initiation of the RMP and subsequent collaborations already represents a first step toward encouraging new malaria prevention behaviour. Community leaders, public health workers and representatives of various organizations working on health-related issues in the area have identified a great need for training and access to up-to-date information and technical support [20].

As a first step, therefore, it needed to be established how much local people understood about the existing malaria problem on Rusinga, assess their socio-economic background and create awareness for the ongoing project while sensitizing community members for active participation. In order to do this, focus group discussions (FGD) and semi-structured individual interviews (knowledge, attitude and practice (KAP) surveys) were carried out to determine socio-economic and behavioural baselines to identify indicators for monitoring programme effectiveness [53], and to reveal the perceptions, misconceptions and practices of malaria control, thus yielding important information needed to plan and revise training activities, develop locally appropriate education material and design effective methods with the ultimate goal to encourage new malaria prevention behaviours.

## Methods

### Study area

Rusinga Island (0°35'–0°44' South; 34°11'–34°22' East; altitude 1,100 m) is 42 km^2 ^in area and is the second largest island in Lake Victoria. Due to its close proximity to the mainland a 200 m long causeway was constructed in 1983 to link the island with Mbita township, the major trading and the administrative centre of the district. The terrain is extensively deforested and generally rocky and hilly with limited vegetation cover. There are a number of seasonal rivers which contain water only during the rainy season and the lake provides the main water source for the population. Two rainy seasons are typical for the area, the 'long rains' between March and June and the 'short rains' between October and November, but these seasons are unreliable with some years characterised by prolonged dry periods. Malaria transmission fluctuates seasonally but is sustained all year round by the three primary malaria vectors: *An. gambiae*,, *Anopheles arabiensis *and, to a lesser extent, *Anopheles funestus *[8,54-56]. As per a census implemented by the end of 2006 during the establishment of a Demographic Surveillance System (DSS), Rusinga island had 24,078 inhabitants which formed 5,425 households and 21% of the population were children below the age of five (S. Kaneko *et al*., Nagasaki University, unpublished data). The predominant language spoken is Dholuo.

People living on Rusinga face a multitude of problems. The island has suffered enormous environmental degradation, soil erosion and extended drought conditions in recent years leaving little productive land and few opportunities to make money other than through fishing. Furthermore, construction activities, deforestation, vegetation clearance and poorly planned infrastructure development has led to an increased abundance of mosquito larval habitats [8], notably those suitable for malaria-transmitting *Anopheles*. The high prevalence of both malaria (50%) and HIV/AIDS (30%) on the island has been a major impediment to socio-economic development [57,58].

Two government health centres serve Rusinga's population; one in the north-eastern part of the Island and one in Mbita township. Additionally, there are three registered and many unregistered private facilities on the island. Due to the bad condition of the roads public transport is rare especially in the rainy season and it is difficult to reach the health facilities.

### The Rusinga Island Child & Family Programme (RICFP)

The RMP is implemented through CCF-Kenya's community-based organization RICFP. The CBO has been carrying out development activities on Rusinga for the past 18 years. CCF is an international NGO with the mission to promote the well-being of children by facilitating quality programmes in education, early childhood development, youth, health and nutrition, all including aspects of environmental health and healthy homes [59]. CCF-Kenya is supported by sponsors for 44,000 individual children from around the world and operates in 30 districts of Kenya. The project involves all families of enrolled children in ongoing activities and decision-making processes. CCF-K affiliate CBOs are owned and managed by parent-committees, selected by neighbourhood groups (*jirani*) of enrolled parents. The project has divided the island in eight administrative zones. Each *jirani *group selects a representative to serve in the parent's committee. Using this system, parents identify their own needs, prioritize them, plan, budget and also implement activities. RICFP reaches a total of 4,352 people in 750 families of which 869 are enrolled children aged below 1 to 17 years through direct sponsorships. RICFP intends to reach the entire Rusinga community with their educational programmes.

### Study design

Prior to the community-wide KAP survey a series of focus group discussions (FGDs) were conducted in all eight administrative zones with eight to 10 community members per zone, selected by RICFP parent committees. Those focus groups contained of equal numbers of members from CCF-enrolled and non-enrolled families and were gender balanced. The topics that were discussed were signs and symptoms of malaria, causes and mode of transmission and prevention of malaria. The information from these discussions was used to guide formulating the questions for a cross-sectional KAP survey which was conducted from April to July 2004. The study adopted the RMP administrative units of zones to implement a stratified random sampling scheme. A two-stage cluster procedure with zones and households within them as the two levels sampling units were adopted. The households were divided into two categories; the CCF-enrolled and the non-CCF-enrolled households. The sample size depended on the number of CCF-enrolled households as the intention was to interview all these households (n = 750). A matching household sample size from the same community was randomly selected from non-CCF-enrolled households. Preference for interview was given to households that had at least one child under five years of age. Therefore, a census was implemented in all the eight zones, recording all families with children under five years of age. From this list, households were randomly selected to match approximately the number of CCF-enrolled households in each zone. A total of 1,500 households were selected for interview.

The study largely used quantitative approaches in data collection through a semi-structured questionnaire consisting of 69 questions, both open ended and closed. While the questionnaire was written in both English and Dholuo, the interviews were conducted in the latter ethnic language.

Pre-tests of the questionnaire were conducted in 14 households and adjustments made accordingly. The interviewers were trained residents of Rusinga Island who were fully familiar with the local language, culture and sensitivities. One questionnaire was administered to one household per compound. Households were defined as a group of individuals sleeping in the same house and depending on the same budget [60]. Because many men on Rusinga are polygamous and live in extended family structures several households usually form a compound and often share in a common pool of resources. For interview, preference was give to the female head of the household because she is typically the care taker of the children and responsible for all household duties. In the event that she was absent at the time of interview either the male head of the household or any child above the age of 12 years was questioned. Interviews were conducted in private to reduce influence of relations. Where occupants were not found on the first visit, two other attempts were made to trace them.

Being part of an integrated development programme at community level, the CCF-enrolled families have been involved in various health training activities including malaria prevention. On the other hand the non-CCF-enrolled families have not been exposed to this type of training and health care. The objective here was to investigate whether there was a measurable difference in knowledge, attitude and practice concerning malaria in families that have been exposed to these activities and been embedded in a social network by an established NGO. In general, the study aimed to investigate whether household socio-economic status or demographic characteristics affect malaria related knowledge, prevention and treatment behaviour and the willingness to participate in a community-based programme. The questionnaire was structured into the following topics: (1) demographics of respondent and household; (2) socio-economic status of the household; (3) knowledge and beliefs about malaria (symptoms, prevention methods, mosquito life cycle) (4) typical practices toward malaria prevention; (5) the treatment-seeking behaviour and household expenditure for malaria treatment; and (6) the willingness to prepare and implement community-based vector control.

### Ethical considerations

The institutional ethical clearance was granted by the joint University of Nairobi – Kenyatta National Hospital ethical review committee (protocol approval number P102/7/2004). In addition, permission was obtained from the district authorities and community leaders. Individual interviews were only started after the purpose of the study had been clearly explained to the participant and an informed consent form was read and signed.

### Data analysis

The semi-structured part of the questionnaire was coded after completion of the survey. All data were entered and analysed using the statistical software package for social sciences (SPSS) Version 14.0. Analyses of the outcome of variables were performed excluding non-responders or missing data points, therefore only valid percentages of the responses were accepted which leads to the fact that the total number of respondents (n) varied between questions. A socio-economic index [61] based on household assets was created using Principal Component Analysis (PCA) [62]. The final PCA was based on 13 asset variables (sofa set, bicycle, radio, television set, solar panel, generator, car battery, mobile phone, boat, fishing net, number of cows, goats and chicken) that were identified by community members during FGDs and explained 27% of the variability in the 13 variables. Each interviewed household was assigned to a socio-economic quintile (most poor; very poor; poor; less poor; least poor) according to PCA. Chi-squared tests (χ^2^) were used to examine whether the distribution of individuals/households among the categories of one variable is independent of their distribution among the categories of the other. Logistic regression analyses (backward stepwise) were used to explain variations in responses to questions about knowledge and behaviour towards malaria and its control (e.g. bednet ownership and bednet use, knowledge of mosquitoes as malaria vector) based on socio-economic and demographic indices.

## Results

### Response rate

Of the 1,500 households selected for the survey residents from 1,451 households were interviewed (97% response rate), 701 households being CCF-enrolled and 750 households being non-CCF-enrolled. Interviewed households were equally distributed over the island with an average of 12.5% (95% C.I. = 10.6–14.4) of all respondents interviewed in each zone. There were 1,054 female and 397 male respondents.

### Socio-demographic characteristics of respondents

The mean age of respondents was 34.5 years (95% C.I. = 33.9–35.2) and 70% were within the age range of 21.5 to 47.5 years. The average household on Rusinga had 6.2 household members (95% C.I. = 6.1–6.3) and 4 children (95% C.I. = 3.9–4.2); 1.7 children below the age of five years (95% C.I. = 1.7–1.8). Nearly all respondents (98%) had lived on Rusinga for most of their life. In 32% of all households, the household head was polygamous with two or more wives. 2% of wives were 18 years or less (n = 23), some as young as 14 years old.

Table 1 shows how some of the demographic and economic variables are distributed in households of different socio-economic level. Notably, the majority of the female headed households were in the poorest socio-economic quintiles. This distribution differs significantly from the male-headed households. Of all respondents, only 21% were educated beyond primary school, among whom 1.6% attained tertiary-level education (Table 2). There was a significant difference in the educational level between women and men, with more men educated beyond primary level and a higher percentage of women without any formal education (Table 2). Unsurprisingly, more highly educated respondents were found in wealthier households (Table 1). CCF-enrolled households belonged predominantly to the lower socio-economic levels including 76% of all respondents without formal education (68 out of 90) and only 4% of respondents had a college degree (one out of 23), indicating that the CCF programme's targeting strategy is well implemented. Interestingly, households of higher socio-economic status were also characterized by a higher number of household members, children and wives. Fishing and small-scale businesses like fish mongering and vegetable sales were the most common income generating activities undertaken by the residents of Rusinga; fishing primarily done by men (62% of male respondents) and small-scale businesses by women (48% of female respondents). A sizeable proportion of women (32%) did not work outside the home (housewives) and depended on the income of their husbands (Table 2). In 30% of households fishing (n = 472) and small-scale businesses (n = 426) were mentioned as main sources of income, respectively. 12% of households (n = 179) received their main income from larger businesses and notably 8% (n = 116) of the households depended primarily on remittances from relatives outside Rusinga (Table 1). Formal employment as major income source was primarily found in households of the highest socio-economic status while most of the labourers for small wage income were found to belong to the poorest households (Table 2). The average monthly budget per household was reported to be Kenya Shillings (KShs) 5,360 (95% C.I. = 5,221–5,505) which equals US Dollars ($) 72.5 (KShs 72 = $ 1) but only 8% of all households (n = 1,312) were able to meet their monthly budget in cash. Notably, the households that were unable to meet their budget were equally distributed over all the socio-economic levels (χ^2 ^= 9.8; d.f. = 4; p = 0.056) indicating that higher socio-economic standard induces higher demand and expectations [60]. The average expenditure that could actually be met by households in terms of cash available was KShs 2,960 (95% C.I. = 2,848–3,068; S.D.: 2,007); 15% of all households lived below the poverty line [6] having less than KShs 1,500 ($ 21) per months. Households of higher socio-economic status met a significantly higher amount of cash per month (Table 1).

**Table 1:** Summary of the socio-demographic characteristics of respondents by socio-economic status

**Characteristics**	**Total N**	**Socio-economic status**				
		
		Most poor (%)	Very poor (%)	Poor (%)	Less poor (%)	Least poor (%)
Total households interviewed	**1451**	19.8	20.2	19.9	20.1	30.0
CCF-enrolled households	**701**	22.0	20.4	20.7	21.7	15.4
*Sex of household head (χ^2 ^= 86.5; d.f. = 4; p < 0.001)*						
male	**1125**	16.1	18.5	19.8	21.4	24.1
female	**326**	32.2	26.1	20.2	15.6	5.8
*Age of respondent (χ^2 ^= 26.5; d.f. = 12; p = 0.009)*						
below 25	**376**	19.9	20.7	17.6	20.2	21.5
25 to 34	**443**	23.7	23.3	17.8	17.2	18.1
35 to 44	**301**	15.0	16.3	23.3	20.9	24.6
above 45	**322**	18.6	19.3	22.4	23.3	16.5
*Educational level of respondent (χ^2 ^= 44.9; d.f. = 12; p < 0.001)*						
none	**89**	24.7	25.8	23.6	19.1	6.7
primary	**1039**	20.8	21.4	19.5	20.1	18.2
secondary	**283**	14.8	14.5	21.6	20.1	29.0
college	**23**	8.7	17.4	4.3	26.1	43.5
*Number of household members (χ^2 ^= 100.0; d.f. = 12; p < 0.001)*						
2 to 4	**384**	27.1	26.3	21.6	14.3	10.7
5 to 7	**685**	21.0	20.1	18.4	18.8	21.6
8 to 10	**299**	10.0	15.4	22.1	29.4	23.1
more than 10	**73**	9.6	9.6	16.4	24.7	39.7
*Number children (χ^2 ^= 49.7; d.f. = 8; p < 0.001)*						
1 to 2	**396**	24.7	24.7	20.7	15.9	13.9
3 to 5	**715**	20.3	21.0	18.7	19.3	20.7
more than 5	**328**	12.8	13.4	21.3	27.1	25.3
*Number of wives of household head (χ^2 ^= 37.3; d.f. = 8; p < 0.001)*						
1 wife	**918**	22.8	21.6	19.9	19.9	15.8
2 wives	**286**	14.3	21.0	20.3	18.9	25.5
more than 2 wives	**154**	15.6	15.6	16.9	19.5	32.5
*Main source of household income (χ^2 ^= 99.1; d.f. = 28; p < 0.001)*						
unskilled labour	**70**	28.6	24.3	30.0	11.4	5.7
skilled labour (craftsperson)	**16**	25.0	25.0	18.8	18.8	12.5
farming	**73**	12.3	17.8	24.7	24.7	20.5
small scale business*	**426**	22.8	23.9	18.3	19.2	15.7
other business	**179**	17.9	21.8	18.4	19.6	22.3
fishing	**472**	17.8	17.6	21.2	22.2	21.2
Salary/pension for employment	**73**	4.1	12.3	16.4	19.2	47.9
support from relative outside Rusinga	**116**	27.6	19.8	12.9	19.8	19.8
*Monthly expenditure met by households(χ^2 ^= 89.5; d.f. = 12; p < 0.001)*						
less than 1500 KShs	**186**	34.9	26.9	17.2	16.1	4.8
1500–3000 KShs	**422**	20.9	23.0	20.9	18.5	16.8
3000–4500 KShs	**434**	15.9	19.6	22.1	20.3	22.1
more than 4500 KShs	**232**	10.3	14.7	19.0	26.7	29.3

* fish mongering & vegetable sales

**Table 2:** Differences in education level and occupational activities between men and women

	**Sex of respondent**	
	
	**male**	**female**
*Education (χ^2 ^= 76.7; d.f. = 3; p < 0.001)*		
Total N	393	1050
none	1.3%	8.1%
primary	64.6%	75.5%
secondary	30.0%	15.7%
college	4.1%	0.7%
*Occupation (χ^2 ^= 800.3; d.f. = 7; p < 0.001)*		
Total N	391	1053
farmer	9.0%	4.6%
labourer	4.6%	2.1%
craftsperson	2.6%	1.1%
fisherfolk	61.6%	2.2%
small-scale business	4.9%	48.0%
business	5.4%	7.8%
formal employment	5.1%	2.1%
none	6.9%	32.2%

### Knowledge and beliefs

**Figure 1:**
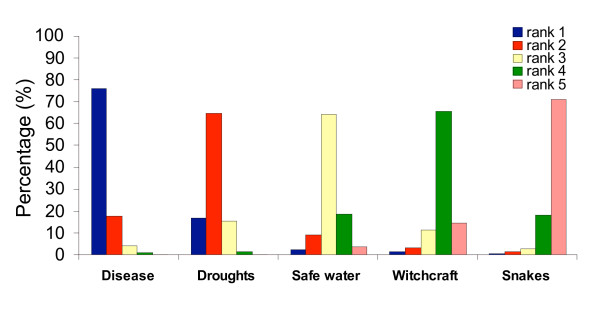
Ranks of perceived major threats of live on Rusinga.

**Figure 2:**
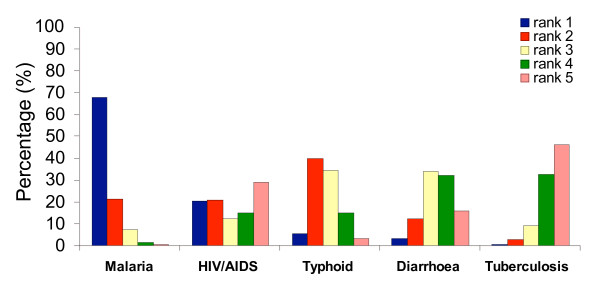
Ranks of perceived importance of diseases on Rusinga.

The respondents were asked about the perceived threats for life on Rusinga, most seriously felt diseases, malaria symptoms, most vulnerable groups to malaria infection, mode of parasite transmission and malaria prevention methods known. In FGDs, community members identified five major threats to life on Rusinga which were: droughts, diseases, no access to safe water, witchcraft and dangerous animals like snakes. For the diseases HIV/AIDS, malaria, diarrhoeal diseases, typhoid and tuberculosis were noted to be most prevalent. In the individual interviews, respondents were requested to rank these threats and diseases in descending order of importance and to name others that might be felt more important (Figure 1 and 2).

Most respondents felt that diseases are the most important threat to life on Rusinga, followed by drought, lack of access to safe water, witchcraft and dangerous animals; 10% of all respondents added famine and 5% added poverty as major threats to the list. Poverty featured in average on rank 5 while famine was ranked 1–3.

Diseases were ranked the most serious problem with 75% of the respondents ranking it first (Figure 1). Among the diseases, 67% of respondents ranked malaria as the most dangerous threat (Figure 2). The ranking of HIV/AIDS, typhoid, diarrhoeal diseases and tuberculosis was less consistent. Notably, there was a very indifferent view about the ranking of HIV/AIDS; only 21% of all respondents ranked it first and second, respectively; 29% of all respondents ranked HIV/AIDS the least important disease threat in comparison to the others. The ranking of life threats and disease importance did not differ with sex, age, CCF-enrolment status or educational level of the respondent.

Over 95% of respondents correctly identified headache, sweating, shivering body, high fever, joint pains, loss of appetite and vomiting as malaria symptoms but 88% of respondents associated malaria also with a running nose, 47% with body rashes and 32% with blood in sputum. Only 9% of all respondents mentioned all malaria symptoms correctly.

Regression analysis revealed that correct knowledge of malaria symptoms was dependent on the education level of the respondent but not the age, sex or CCF-enrolment. Respondents educated beyond primary school level were far more likely to correctly list the malaria symptoms (Table 3).

**Table 3:** Factors associated with correct biomedical knowledge of malaria symptoms

Education*	**Odds Ratio**	**95% C.I. for Odds Ratio**		***P***
			
		**Lower**	**Upper**	
none	1.000			
primary	1.248	0.491	3.175	0.641
secondary	2.707	1.033	7.096	0.043
college	10.800	3.154	36.984	<0.001

*Variables entered on step 1: sex, age group, CCF-enrolment and education level of respondent

Among respondents, 96% knew young children to be at highest risk of suffering from severe malaria and 76% also knew of pregnant women being at increased risk. But 60% and 24% of the respondents also believed that young adults and old people were at high risk of suffering severe malaria, respectively. Other categories of people mentioned were fishermen, those who do not use bednets, tourists, dirty people and those who do not eat a balanced diet.

Of all respondents (n = 1,445), 5% did not know what caused malaria. Of those who indicated they knew (n = 1,378), 91% mentioned mosquito bites but more importantly only 47% correctly knew that was the only route for malaria transmission. A large proportion (44%) of the community believed in a number of causes in addition to mosquito bites and 9% of all respondents did not mention mosquitoes at all. Other major reasons believed to be responsible for *'catching malaria' *(Table 4) were unfavourable weather conditions (cold temperatures, change of weather from cold to hot or *vice versa*, when one is rained on, sitting in the sun for too long, at times of new moon), lack of hygiene (drinking of dirty water, walking barefoot in dirty environment, unhygienic conditions at home, badly ventilated house, dirty utensils, dust, lack of a latrine or rubbish pit), a bushy compound (planting crops next to the house, bushes and high grass on the compound), food (raw, cold, contaminated or processed food) and body exhaustion (hard labour, no sleep, starving, fever).

**Table 4:** Respondents' believed causes of malaria

**Causes of malaria**	**n**	**%**
*Total N = 1378*		
Mosquito bites	1250	90.7
Weather conditions	423	30.7
Lack of hygiene	250	18.1
Bushy compound	156	11.3
Food	69	4.8
Body exhaustion	31	2.1

Logistic regression analyses were used to identify the variables impacting correct knowledge and misconceptions/traditional beliefs (Table 5). Education beyond primary school level increased the probability of respondents knowing mosquito bites as the sole cause of malaria by 3–4 times.

**Table 5:** Demographic variables impacting correct knowledge and misconceptions on what causes malaria

Variables*	**Odds Ratio**	**95% C.I. for Odds Ratio**		***p***
			
		**Lower**	**Upper**	
*Factors associated with knowledge of mosquito bites as sole cause of malaria*				
Education*				
none	1.000			
primary	1.473	0.910	2.385	0.115
secondary	2.837	1.681	4.787	<0.001
college	4.400	1.615	11.990	0.004
*Factors associated with belief that unfavourable weather causes malaria*				
Non-CCF*	1.488	1.172	1.890	0.001
Education*				
none	1.000			
primary	0.871	0.536	1.416	0.578
secondary	0.287	0.162	0.510	<0.001
college	0.204	0.055	0.756	0.017
*Factors associated with belief that lack of hygiene causes malaria*				
Age*				
below 25	1.000			
25 to 34	0.985	0.666	1.457	0.939
35 to 44	1.175	0.775	1.783	0.448
above 45	1.926	1.309	2.834	0.001
*Factors associated with not mentioning mosquito bites as cause of malaria*				
Non-CCF*	2.309	1.483	4.209	<0.001
Age*				
below 25	1.000			
25 to 34	1.113	0.659	1.880	0.690
35 to 44	1.250	0.703	2.222	0.448
above 45	2.694	1.578	4.600	<0.001

*Variables entered on step 1: sex, age group, CCF-enrolment and education level of respondent

Conversely, community members educated beyond primary school level were less likely to believe that unfavourable weather conditions was responsible for catching malaria. Older age groups were more likely to believe that malaria is caused by unhygienic living conditions and were also more likely not to mention mosquitoes involved in malaria transmission at all. Non-CCF-enrolled community members were less likely to mention mosquitoes and more likely to believe in weather as a cause of malaria than CCF-affiliated community members. There were no differences in knowledge and beliefs between men and women.

Table 6 illustrates how, according to community members, malaria parasites enter the human body. A large proportion (61.8%) of the respondents (n = 1,439) stated mosquito bites only; 7.6% believed in other ways in addition to mosquito bites, while 10.4% thought that mosquitoes had no role in the parasite transmission.

**Table 6:** Respondents' believed way of parasite infection

**Ways of parasite entry**	**n**	**%**
*Total N = 1439*		
Mosquito bites	999	69.4
Dirty water or food	138	9.6
Body openings & cuts	125	8.7
Don't know	291	20.2

Notably, 20.2% of all respondents declared they do not know; this included respondents that mentioned mosquitoes as cause of malaria earlier. Other beliefs on how one could get infected with malaria parasites included consuming dirty water and food, or through cuts in the skin, and through ears and mouth. Of all respondent that knew that the malaria parasites can only enter the human body through mosquito bites (n = 890), 50% stated unfavourable hygienic and weather conditions as causes of malaria earlier. More surprisingly, more than half of the respondents that did not cite mosquitoes as cause of malaria earlier identified mosquito bites as the only way that malaria parasites can enter the human body indicating that despite the fact that there is a lot of knowledge in the community, this knowledge is distorted and biomedical relationships not comprehended.

Although over 60% of the interviewees correctly stated that mosquito bites are responsible for injecting the malaria parasite into the human body, none of the respondents was able to explain correctly where this parasite has been picked up by the mosquito. A common belief was that the parasite comes from various sources of water, bushes, dirty environments, food and air indicating that the cycle of malaria transmission via mosquitoes from a sick person to a healthy one has not been comprehended at all.

Evaluating the community knowledge of the mosquito life cycle 87% of the respondents (1,254/1,451) said they knew where mosquitoes lay their eggs. Places mentioned included stagnant water (79%), bushes and grass (6%), humid places (4%) and dark corners (3%). The majority (65%) of respondents did not know what mosquito larvae look like and those (35%) who attempted to define them talked of worm-like invertebrates, small toads, small insects, animals with big heads and small abdomens as well as twinkling reflections in water.

Knowledge of malaria prevention methods differed only slightly between households. Most respondents mentioned more than one method known to them as shown in Table 7; only 4% of all respondents stated not to know at all how to prevent malaria.

**Table 7:** Respondents' believed/known and used methods for malaria prevention

**Methods**	**Methods known**		**Methods used**		χ^2^	p*
			
	**N**	**%**	**N**	**%**		
*Total N = 1451*						
Bednets	1274	87.8	692	47.7	528.0	<0.001
Bush clearing	633	43.6	492	33.9	28.4	<0.001
Destruction of burrow pits that can collect water	492	33.9	0	0.0	592.4	<0.001
Burning/spraying insecticide or mosquito repellents	233	16.1	236	16.3	0.1	*ns*
Boiling/treating water	186	12.8	109	7.5	22.4	<0.001
Taking anti-malarial drugs	183	12.6	170	11.7	0.5	*ns*
Keeping body/food warm and clean	170	11.7	0	0.0	180.6	<0.001
Proper disposal of empty tins that can hold water	122	8.4	309	21.3	95.3	<0.001
Keeping utensils, house and compound clean	117	8.1	38	2.6	42.5	<0.001
Burning rubbish in the compound	77	5.3	42	2.9	10.7	0.010
Use traditional herbs	48	3.3	61	4.2	1.6	*ns*
None	52	3.6	184	12.7	64.5	<0.001

*d.f. = 1; for significance *p *> 0.05 chi square (χ^2^) should be ≥3.84.

The majority of interviewees had a good theoretical knowledge of how to prevent malaria, with bednets most frequently mentioned. A summary of all methods which community members believed to be useful for preventing malaria is given in Table 7. Notably, clearing vegetation was the second most common method which community members believed to be useful to prevent malaria despite the fact that it is established knowledge in the scientific community that clearing of vegetation is of no benefit but might even worsen the malaria situation [63-67].

Of those that responded to know how to prevent malaria (n = 1,398) only 34% listed solely biomedical correct methods to target either the mosquito adults (bednets, repellents), larvae (destruction of holes with stagnant water) or malaria parasite (drugs); but 72% listed only correct prevention methods with the one addition of bush clearing. Another 25% mixed correct knowledge and beliefs e.g. that increased hygiene, disposal of rubbish or keeping warm would help to prevent malaria. Only 3% of all respondents did not list any scientifically correct malaria prevention measure.

The probability of knowing that bednets could be used for malaria prevention was significantly higher in CCF-enrolled families than non-CCF families and also increased with increased levels of education. Moreover, people more than 45 years old were less likely to mention bednets than younger people (Table 8).

**Table 8:** Demographic variables impacting correct knowledge and misconceptions about potential methods to prevent malaria

Variables*	**Odds Ratio**	**95% C.I. for Odds Ratio**		***p***
			
		**Lower**	**Upper**	
*Factors associated with knowledge of bednets*				
Non-CCF*	0.438	0.306	0.627	<0.001
Age*				
below 25	1.000			
25 to 34	1.393	0.882	2.200	0.155
35 to 44	1.164	0.703	1.926	0.555
above 45	0.525	0.327	0.844	0.008
Education*				
none	1.000			
primary	2.071	1.179	3.638	0.011
secondary	3.928	1.935	7.975	<0.001
college	542.724	0.000	1.43E+09	0.404
*Factors associated with knowledge of source reduction (filling burrow pits)*				
Non-CCF*	0.712	0.566	0.894	0.004
Women*	0.745	0.579	0.959	0.022
Education*				
none	1.000			
primary	1.032	0.637	1.672	0.899
secondary	2.660	1.569	4.510	<0.001
college	7.446	2.570	21.573	<0.001
*Factors associated with knowledge of insecticides and repellents*				
Non-CCF*	0.514	0.381	0.693	<0.001
Age*				
below 25	1.000			
25 to 34	0.937	0.653	1.343	0.723
35 to 44	0.542	0.349	0.842	0.006
above 45	0.576	0.376	0.883	0.011
*Factors associated with belief in bush clearing*				
Non-CCF*	0.552	0.445	0.685	<0.001
Women*	0.736	0.578	0.938	0.013
Education*				
none	1.000			
primary	1.602	1.007	2.551	0.047
secondary	2.431	1.454	4.067	0.001
college	3.312	1.263	8.689	0.015
*Factors associated with belief in increased hygiene and keeping warm*				
Non-CCF*	1.419	1.107	1.818	0.006
Age*				
below 25	1.000			
25 to 34	1.279	0.929	1.762	0.132
35 to 44	1.335	0.937	1.903	0.110
above 45	1.639	1.132	2.371	0.009
Education*				
none	1.000			
primary	0.639	0.394	1.037	0.070
secondary	0.544	0.316	0.937	0.028
college	0.205	0.056	0.758	0.018

*Variables entered on step 1: sex, age group, CCF-enrolment and education level of respondent

Source reduction was less likely to be mentioned by non-CCF affiliated community members and women compared to CCF-enrolled members and men, respectively, furthermore it was 3–7 times more commonly mentioned by respondents educated beyond primary level.

Whether respondents mentioned repellents and insecticide use for malaria prevention was confounded by their CCF enrolment status and age. The methods were less likely to be mentioned by respondents not affiliated to CCF and older age groups. Interestingly, the belief that bush clearing can prevent malaria was twice as high in families that were enrolled in CCF compared to non-CCF respondents and more importantly increased significantly with the higher the level of education. Women were less likely to mention bush clearing than men. The probability of believing in measures of general hygiene for malaria prevention increased with being a non-CCF-enrolled respondent and with older age but decreased with education beyond primary level.

### Malaria prevention behaviour

The actual malaria prevention behaviour practiced by community members differed significantly from the methods known to respondents (Table 7). Despite the fact that 88% of all interviewees knew that bednets protect from malaria only 58% used them and in only 48% (692/1,451) of the interviewed households could the ownership of one or more nets actually be confirmed. Furthermore, only 37% of respondents (535/1,451) slept under a bednet the night before the interview, which was held during the main malaria transmission season. Of those, most (94%) said they sleep under a net throughout the year, while a few only used a net when mosquitoes are abundant. Children were the main beneficiaries of bednets: In 88% of the net-owning households children slept either alone or with their parents under the bednet. In total, 1,073 bednets were found in 692 households serving 4,419 people. This accounts for 1.5 bednets per household or 0.2 bednets per person in bednet-owning households alone.

Consequently, the average bednet coverage for the entire community was 0.7 bednets per household or 0.1 bednet per person which is not enough for a community-level effect [68]. Remarkably, of those respondents that correctly identified mosquito bites as the only means of malaria transmission (n = 642), 48% did not own a mosquito net while, in contrast, 40% of those that did not associate malaria with mosquito bites (n = 128) owned a net indicating that net ownership does depend on socio-demographic factors other than knowledge alone.

Bush clearing was the second most common method that community members practiced (492/1,451); 51% defined bush clearing as cutting down all the vegetation on the compound and burning it, 46% only slashed grass and 3% characterised it as collecting vegetation and empty containers from the surroundings. 56% of respondents said they cleared the bush in the last month. The activity was predominantly implemented by men. Of the 492 respondents that practiced bush clearing, 20% believed that bushes and other vegetation served as larval habitats for mosquitoes and 80% believed that mosquitoes hide in vegetation and can be prevented from entering the compound by removing it. Most (70%) said they learned that clearing vegetation prevents malaria in school. Another important source of this information was national and international health care organizations like local extension workers of the Ministry of Health and NGOs, including CCF and UNICEF. Equally worrying, radio and newspaper announcements on malaria control were also cited.

Despite that there was a very good knowledge in the community that the removal of water containing borrow pits protects from malaria, a fact recently highlighted with particular strength in nearby areas [69] nobody actually practiced this. Notably, 13% of all respondents did nothing to prevent malaria.

While knowledge of prevention measures might be confounded by demographic variables like the age and education level of the respondent, the actual usage of various methods might be decided by the household head and depend on the socio-economic status of the household [70]. The results of logistic regression analyses to investigate this are summarised in Table 9. Socio-economic status had significant impact on the probability of a household owning a bednet, boiling drinking water or not practice any prevention behaviour. Wealthier households were more likely to practice malaria prevention than poorer ones, thus they were more likely to own a bednet and buy fuel for boiling water. Insecticide use, like burning mosquito coils, was independent of the socio-economic status but women-headed households were more likely to practice this. Proper disposal of tins and rubbish was more likely to be practiced by men-headed households. Clearing vegetation in and around the compound was expectedly independent of the socio-economic status of the household but was more likely to be practiced by CCF-enrolled than non-enrolled families and men-headed households than women-headed households. Use of anti-malaria drugs was independent of CCF enrolment status, sex of household head and socio-economic status of the household.

**Table 9:** Socio-economic variables impacting malaria prevention behaviour

Variables*	**Odds Ratio**	**95% C.I. for Odds Ratio**		***P***
			
		**Lower**	**Upper**	
*Factors associated with ownership and usage of bednets*				
Socio-economic status*				
most poor	1.000			
very poor	1.918	1.186	3.101	0.008
poor	3.816	2.241	6.498	<0.001
less poor	3.640	2.205	6.008	<0.001
least poor	6.849	3.885	12.076	<0.001
*Factors associated with use of insecticides and repellents*				
Women-headed*	1.470	1.073	2.014	0.017
*Factors associated with proper disposal of tins and rubbish*				
Women-headed*	0.708	0.514	0.974	0.034
*Factors associated with bush clearing*				
Non-CCF*	0.687	0.547	0.864	0.001
Women-headed*	0.564	0.424	0.751	<0.001
*Factors associated with boiling/treating drinking water*				
Women-headed*	1.929	1.240	3.000	0.004
Socio-economic status*				
most poor	1.000			
very poor	1.807	0.889	3.673	0.102
poor	2.315	1.157	4.632	0.018
less poor	2.384	1.187	4.791	0.015
least poor	2.185	1.053	4.534	0.036
*Factors associated with not practicing any malaria prevention behaviour*				
Non-CCF*	1.400	1.001	1.958	0.049
Women-headed*	1.461	1.000	2.134	0.050
Socio-economic status*				
most poor	1.000			
very poor	0.607	0.383	0.961	0.033
poor	0.699	0.444	1.099	0.121
less poor	0.430	0.258	0.717	0.001
least poor	0.469	0.282	0.781	0.004

*Variables entered on step 1: CCF-enrolment, sex of household head, socio-economic status

### Malaria treatment seeking behaviour and associated expenses

There was a pronounced difference between the theoretical and practical malaria treatment seeking behaviour of community members. In response to the theoretical question *'what do you do if you suspect you or a member of your family has malaria?' *44% (n = 641) of interviewees stated they would go to hospital, 54% would buy drugs or prepare traditional herbs first and go to hospital only if the patient does not get better. One percent made the decision dependent on the availability of cash, if money would be available advice would be sought at hospital otherwise drugs would be bought at a local shop. A few people preferred to pray.

Of all interviewed households (n = 1,451), 68% reported a child below the age of five years being sick in the last two weeks. The total expenditure for treating sick children ranged from KShs 2 to 10,000 with a median of KShs 250 (IQR 400). Of the occurring costs, 92% were spent on drugs and only 5.5% on laboratory and doctors' fees and 2% on transport indicating an even lower frequentation of health facilities in practice compared to theory. The remaining 0.5% was spent on herbalists and traditional herbs. 82% of respondents with a sick child felt that the disease had negative impacts on their daily life. Women were specifically concerned that they could not run their small-scale businesses and had to neglect farming and domestic work in order to take care of the child. There was no difference in health-seeking behaviour in relation to CCF-enrolment status, education level of respondents or socio-economic status of the households.

### The role of the community in malaria control

Most interviewees (939/1,430; 64%) said that malaria could be controlled on Rusinga island, while 34% (491/1,430) did not believe so, with 2% were undecided. Table 10 summarizes the reasons given for not being able to control malaria. Most of these were based on misconceptions or a fatalistic attitude. With increasing education level respondents were more likely to believe that malaria can be successfully controlled on Rusinga (Table 11).

**Table 10:** Community's reasons to believe that malaria can not be controlled

**Reasons**	**n**	**%**
*Total N = 491*		
Malaria is a God given fact of life	141	28.7
Impossible to kill all the mosquitoes	125	25.5
People can not afford prevention and treatment	60	12.2
People do not take preventive measures	53	10.8
We are surrounded by the lake (believed breeding habitat)	48	9.8
Malaria multiplies fast and can't be stopped	23	4.7
It is a virus which can not be stopped	9	1.8
There is a lot of dust on Rusinga causing respiratory illness and malaria	4	0.8
No reason	28	5.7

**Table 11:** Factors associated with belief that malaria can be successfully controlled on Rusinga

Education*	**Odds Ratio**	**95% C.I. for Odds Ratio**		***P***
			
		**Lower**	**Upper**	
none	1.000			
primary	1.619	1.048	2.499	0.037
secondary	2.878	1.754	4.722	<0.001
college	4.644	1.463	14.749	0.010

*Variables entered on step 1: sex, age group, CCF-enrolment, education and socioeconomic status

A number of suggestions were made by respondents who believed that malaria control could be successful on Rusinga (n = 939) as to what needs to be done (Table 12). People most frequently mentioned that everybody needed to sleep under a mosquito net. This was closely followed by the belief that for successful control everybody on Rusinga would need to clear the vegetation from the compounds and thirdly the drainage of stagnant water and the treatment of larval habitats was mentioned.

**Table 12:** Community's opinion of what needs to be done to control malaria on Rusinga

**Reasons**	**n**	**%**
*Total N = 939*		
Everybody needs to sleep under a bednet	482	51.3
Everybody needs to clear vegetation for compound to be open	346	36.8
Stagnant water needs to be drained or treated to kill larvae	258	27.5
Government to provide control (including free bednets, larvicide and clearing vegetation)	91	9.7
Everybody to keep compound clean and built latrines	84	8.9
Free and proper diagnosis and treatment in hospitals	79	8.4
Everybody to take anti-malarial drugs	57	6.1
Provision of training on malaria control	52	5.5
Provision of access to safe water	33	3.5
Everybody to use preventive measures in the house	27	2.9
Malaria vaccine needs to be introduced	12	1.3
Living standards need to be improved	3	0.3

Although a large number of community members thought that malaria can be controlled, there seemed to be little that the community felt they could contribute personally and without outside assistance. Moreover, the majority of contributions that were suggested would not even target malaria control (e.g. clearing vegetation, teaching to boil water, collect rubbish, constructing latrines; Table 13). A total of 12% (n = 243) felt they can not personally contribute at all. The most frequently associated benefit with malaria control was that the family would be happier because no-one would be sick and that time and money could be saved for other *'family projects'*.

**Table 13:** Responses to 'How can YOU contribute to malaria control on Rusinga?'

**Contributions**	**n**	**%**
*Total N = 939*		
Personal oriented protection (bednet, insecticides, repellents)	243	25.9
If I would be trained I could teach people	170	18.1
I can clear vegetation around my house	166	17.7
I can advise people to clear vegetation	153	16.3
I can teach people to boil water	72	7.7
I can participate in removal of empty containers	68	7.2
I would actively participate in a program that tells me what to do	53	5.7
I can spay paraffin on waterholes	5	0.5
I can buy medicines and bednets for people	4	0.4
I can help constructing latrines	3	0.3
I can only contribute if I am given money	3	0.3
I can not contribute	113	12.0
I do not know how I could contribute	45	4.8

Community members expressed the need to be properly trained on how to prevent malaria during the FGDs and the individual interviews. Only 12.3% of all interviewees remember receiving malaria-related health education in the past, with CCF being the most frequently-mentioned training partner. In fact trained respondents were twice as likely to have been CCF-enrolled than non-enrolled community members (Odds ratio = 0.493 (for non-CCF), 95% C.I. = 0.356–0.682, d.f. = 1, p < 0.001). Other training partners mentioned were the Ministry of Health, churches, schools, the media and local CBOs. Major training components were general cleanliness, collecting tins, clearing vegetation and the use of bednets. Some of those trained said the training had helped them in different ways by encouraging them to drain stagnant water, buy mosquito nets and self-medicate. Others however, said they had financial constraints and had not implemented what they were taught. 72% of respondents were aware of the malaria project initiated by the RICFP but did not exactly know its goals; the majority demanded to be given free bednets and malaria medication through the project.

## Discussion

The most striking results of the community survey were that malaria is considered one of the major threats to life but that local knowledge about malaria transmission is a chimera of scientific knowledge (e.g. anopheline mosquitoes transmit the disease) and local beliefs (e.g. being rained on, eating sugar cane, or lack of hygiene cause malaria) which combined with impoverishment leads to ineffective malaria prevention. Misconceptions relating to malaria found in this study were remarkably similar to those found elsewhere in Africa [70-75] and other parts of the world [26,76,77]. The findings show that there is a high level of what community members term as *'western knowledge' *in the Rusinga community which is not completely trusted and, therefore, high priority is still given to traditional beliefs. In a FGD one female participant stated *'my son has malaria and I believe it is because of the cold and wet weather'*. When reminded that only mosquitoes transmit malaria she shook her head and replied *'I have bought a mosquito net for my son and he sleeps under it every night but he was diagnosed with malaria' *indicating her scepticism based on the fact that she uses a bednet, but malaria was not prevented as promised. This lack of trust in health messages from *'outside' *has been frequently expressed in African communities [70,74,78]. Furthermore, a number of contradicting responses during our survey indicate that despite the fact that there is a lot of knowledge in the community, this knowledge was distorted and causal connections were not understood raising questions about the quality of past health education messages and whether they might be more confusing than helpful if not implemented in a cultural sensitive way.

Although many (88%) knew bednets prevent malaria, only 48% of households actually owned a net, with only 37% sleeping under one the previous night. In comparison with other African communities at the time of the survey the bednet coverage was moderate [79]. In contrast to other studies [79] though Rusinga's community had a very high knowledge of children being the most vulnerable to severe malaria and an extremely high coverage of children in bednet-possessing households. This provides an excellent base for increased training on bednet use and availability of nets to protect the target population and reach the Abuja target [80]. In agreement with other studies [75,81,82] bednet ownership was primarily dependent on the socio-economic status of the household. They are luxury assets which are there in better-off households even if the family might have limited knowledge on malaria. Despite Rusinga being a very poor community, a relatively low percentage lived below the poverty line (15%) as compared with the entire country (an estimated 50% of rural dwellers live below the poverty line [6,83]). This might be explained by the fact that the majority of inhabitants of Rusinga make a living by trading in fish. It has been shown that fishing families tend to be better-off than their purely farming counterparts in rural areas of western Kenya [83]. Taking these facts into account improving availability of bednets and selling them at reduced costs should help to quickly improve household coverage on Rusinga. During the individual interviews no question was specifically asked about insecticide treatment of the nets. Interestingly though, none of the respondents mentioned insecticide treatment of nets as a malaria prevention measure. During FGDs, most participants were unfamiliar with insecticide treatment of nets (less than 10% of bednets owners knew their bednet to be treated) and the majority did not know what it would be good for. One FGD with 14 participants was specifically implemented to discuss bednet use and insecticide treatment, and only 2 participants said that insecticide treatment of nets repels mosquitoes from the house. Nobody knew that treated nets kill mosquitoes. The observation that there was little understanding in the community about insecticide treatment of nets despite the fact that there was good knowledge of bednets as a malaria prevention measure and moderate bednet coverage has been frequently reported [70,71] and indicates a gap in explaining to community members the causal connections to comprehend and implement the method.

CCF enrolment did not particularly decrease potential vulnerability to malaria, in most cases the knowledge, attitudes and practices in households were not different from those non-enrolled.

Nevertheless, CCF-enrolled families had better knowledge of bednets which is mainly attributed to the fact that CCF had distributed free nets to their families in the past. CCF households were also more aware of stagnant water as mosquito breeding sites but, like others, they did not practice any behaviour to prevent these sites. CCF-enrolment most strikingly increased the implementation of bush clearing for malaria control. In fact, apart from bednet use, the major activity implemented by community members to prevent malaria in Rusinga, as reported elsewhere [73,75], was the clearing of grass and bushes around the compounds and other general sanitation and hygienic measures (e.g. collection of small containers), none of which have a proven efficacy to prevent malaria in Africa. Teaching evidence-based methods needs to take the local ecology of mosquitoes into account [52,84-86] because methods which are appropriate in one area might not be in another. While vegetation clearance can help control shade loving malaria vectors it will increase the abundance of *An. gambiae *in East Africa which prefers open sun exposed habitats [10,87-90]. That the clearing of bushes removes mosquito adult resting sites and consequently helps prevent malaria has never been proven and is considered ineffective [63-66,91]. *An. gambiae *is highly anthropophilic and rests primarily inside houses. Outside shelters are difficult to find and are rarely bushes or grass but rather rocks or other artificial shelters like granaries [92,93] which are very darkly shaded [94].

Despite the scientific evidence, bush clearing for malaria control remains *'a remarkably widespread and persistent myth' *[64]. Misleading health education messages for community based malaria control can be found for example in the training guidelines of CCF [95] or UNICEF [96] and even in primary and secondary school books [97-101] which explains the significantly increased knowledge and practice of this methods in CCF-enrolled and well educated people. Cutting of bushes and grasses around the house and the removal/emptying of small containers from the environment are the primary messages despite none of them serving malaria vectors as oviposition or resting sites. The common application of these practices might be explained by the fact that they do not need any resources and might therefore be dictated by circumstances [73]. Consequently, community based efforts often do not actually target what they intend to do at the outset and changes in educational strategies are necessary to achieve evidence-based malaria prevention behaviour at the community level. More interdisciplinary collaboration between socio-behavioural scientists, education specialists and entomologists would be desirable for designing evidence-based and culturally sensitive interventions and to avoid confusing reports even from the scientific literature [32,45,102,103].

The community hardly distinguished between malaria and other diseases and most illnesses are referred to as malaria. Obvious confusion could be observed with TB and HIV/AIDS as shown by the frequent mention of blood in sputum as a malaria symptom and the fact that 60% of respondents felt that young adults are at major risk of severe malaria which is most likely associated with the high mortality rate of this age group due to AIDS. Most of the community reacted to illness with self-treatment, which is found in most African communities [73,74,104-106]. Treatment is mainly done with modern and rarely with traditional medicine which provides opportunities for improvement of drug use through shop keepers training and training on home-based treatment. Most cases of illness are not attended by professional health personnel due to the inaccessibility of health centres and associated costs. This has implications for the impact of national malaria control strategies which mainly target improvement of diagnoses and drugs in government health facilities.

Age and education have been the prime factors responsible for a good knowledge and behaviour. Local beliefs and misconceptions were more frequently expressed by older people. With increase in education, this problem could be overcome given that health messages are developed in a way that they can be trusted by the community. Women are typically the major care givers in the family but were often very poorly educated. Men are in most cases the household heads and decide on how the family resources are spent [70]. Therefore, programmes need to target women and men alike. It is important to note, that the probability of having a better understanding of malaria transmission and control was only significantly increased when the respondents education level went beyond the primary school level. This indicates limited contributions of primary education, which is the major level of education found in the community. In fact, a FGD with primary school teachers revealed basically the same knowledge and misconceptions as the rest of the community (see also [107]). Therefore, primary school teachers need to be included and targeted by the RMP to improve their knowledge to consequently improve school health education. Schools are an important entry point for malaria education [47,107,108] and in a country with over seven million primary school children they present a great opportunity to improve health in the community [32]. The issue of vegetation clearance is currently an examination topic at Kenyan schools, constituting a major problem which urgently needs to be corrected. This calls for the national curriculum reform and retraining of extension workers and revision of standard teaching aids used in schools.

Training in support of malaria control needs to aim at an integrated disease management, even at the individual level. Explanations need to be provided to local communities of causal connections in malaria transmission and control to increase the trust in methodologies. It is important for lay people to understand that a bednet gives you extremely high protection, even more so when insecticide-treated, but can not protect somebody 100% [109]. Therefore, the implementation of additional evidence-based control methods is vital, like the drying or covering (e.g. through planting of trees and tall reeds) of stagnant water or the mosquito proofing of houses. The latter is a well known and promising method for malaria control [110] which has been conspicuously absent from peoples mind.

The majority of respondents thought that malaria could be controlled on Rusinga Island, and would be willing to contribute in various ways to a community-based malaria control programme. However, they feel that they do need outside assistance which calls for a programme that does encourage and enable the community through evidence based, participatory learning and involvement in programme implementation.

The possible origin of various beliefs and misconceptions about malaria transmission and prevention were investigated during the FGDs in order to find out where they might originate from or how they can be connected to the biomedical context of malaria transmission. The community identified two major groups of beliefs or misconceptions: 1) issues to do with general hygiene and 2) weather conditions and certain type of food. It was discussed that malaria is often a synonym for all type of illnesses in the community and that most health education messages focus on general hygienic conditions like boiling of drinking water, use of latrine and keeping the compound free of rubbish. Since malaria symptoms were felt not to easily distinguish from those of other diseases these general prevention measures of disease were applied to malaria as any other. Furthermore, FGDs and the interviews revealed that despite the relatively high level of awareness that mosquitoes are involved in malaria transmission there was no knowledge at all as to where the malaria parasite gets picked up, which is where unhygienic living conditions serve as an explanation. The second group of believes was discussed after establishing the seasonality of malaria transmission on Rusinga from appearance of stagnant water (larval habitats), to the highest nuisance mosquito biting and the time when most families have sick children in their home. The community members came to realise that most beliefs are associated with the end of the rainy season and beginning of dry season. This is the time when there is considerable rain and sudden changes of weather from cold to hot or *vice versa*, when people do a lot of physical labour in the fields and when certain crops or fruits are available; but it is also the time of highest biting rates and increasing malaria prevalence. It will, therefore, be useful to include these beliefs in health training activities and develop satisfactory explanations with the community which might lead to a better understanding and higher acceptance of the *'western knowledge' *after all.

## Conclusion

There is an urgent need to design culturally sensitive but evidence-based education interventions which take local beliefs into account and which help the community to understand the causal connections between mosquito habitats, malaria transmission, malaria symptoms, treatment and prevention. The authors hypothesize that this will be best achieved through participatory, '*hands-on experience' *[52], including the community in mapping of larval habitats, studying the mosquito life cycle by rearing them, collecting adult mosquitoes in houses, implementing various vector control strategies and monitoring their impact. Similar approaches have proven highly successful [52] leading to improved malaria prevention behaviour and a decrease in the implementation of inadequate or even exacerbating measures. NGOs, central government education departments and schools have a vital role to play in enabling communities to access appropriate information but need to be equipped with essential knowledge and expert support which could be gained by establishing partnerships with national and international research and tertiary education institutions so that evidence-based research can be applied at the grassroots level [20].

## Authors' contributions

All authors were involved in the design of the study. PO and IK were responsible for the implementation of the study in the field. PO coded the data and supervised data entry. UF and GK analysed the data. UF and PO wrote the first draft of the manuscript. All authors were involved in finalising the manuscript, and read and approved the final version.
